# Familial adult-onset chronic idiopathic megacolon: diagnostic and surgical challenges—a case report

**DOI:** 10.1093/jscr/rjaf1037

**Published:** 2026-01-31

**Authors:** Mohamad Shbaro, Bassel Hafez, Samer Doughan

**Affiliations:** Faculty of Medicine, American University of Beirut, PO Box 11-0236, Riad El Solh, Beirut 1107 2020, Lebanon; Division of General Surgery, Department of Surgery, American University of Beirut Medical Center, PO Box 11-0236, Riad El Solh, Beirut 1107 2020, Lebanon; Division of General Surgery, Department of Surgery, American University of Beirut Medical Center, PO Box 11-0236, Riad El Solh, Beirut 1107 2020, Lebanon

**Keywords:** case reports, colectomy, colonic pseudo-obstruction, gastrointestinal motility, genetic predisposition to disease

## Abstract

Chronic idiopathic megacolon is a rare disorder characterized by persistent colonic dilatation without obstruction or secondary causes. It is usually diagnosed in childhood, often linked to Hirschsprung’s disease; adult-onset cases are exceedingly rare, especially with familial predisposition. We report a man in his 30s with progressive abdominal distension, prior adolescent colectomy, and family history suggesting genetic susceptibility. After excluding secondary causes, chronic idiopathic megacolon was diagnosed. Conservative management failed, necessitating subtotal colectomy with ileorectal anastomosis, leading to complete symptom resolution. Histopathology confirmed normal ganglion cells. Adult-onset idiopathic megacolon poses diagnostic and therapeutic challenges and may have a genetic basis. Subtotal colectomy is safe and effective in refractory cases.

## Introduction

Chronic megacolon is a rare gastrointestinal motility disorder characterized by persistent colonic dilatation or increased compliance in the absence of mechanical obstruction or secondary causes [1]. Patients typically present with refractory constipation, abdominal distention, bloating, and pain, which significantly impair quality of life [2].

While most cases occur in neonates and are associated with Hirschsprung’s disease or MEN2B syndrome [3], adult-onset chronic megacolon is uncommon. Adult presentations are often acute—such as Ogilvie syndrome or toxic megacolon [4, 5]—whereas chronic forms are usually idiopathic [1]. Some cases are associated with systemic diseases, including Duchene’s muscular dystrophy, visceral myopathy, Chaga’s disease, Parkinson’s disease, Ehlers–Danlos syndrome, amyloidosis, and scleroderma [1]. Although most adult cases are sporadic [3], familial clustering suggests a genetic or neuropathic basis [6–8].

Diagnosis of idiopathic megacolon is clinical and radiological, requiring exclusion of secondary causes [9]. Delayed diagnosis may lead to progressive colonic dilatation, pseudo-obstructions, malnutrition, and bowel perforation [6]. Initial management is conservative with dietary modifications, laxatives, and prokinetics [1, 10], reserving surgery for refractory or severe cases.

We present the case of an adult-onset chronic idiopathic megacolon in a patient with prior colectomy in adolescence and a family history suggestive of a heritable predisposition, successfully managed with subtotal colectomy with ileorectal anastomosis. This report follows the SCARE criteria [11].

## Case presentation

A male in his 30s presented to clinic with intermittent abdominal pain, progressive distension, bloating, nausea, reduced oral intake, and recent-onset obstipation. He described similar episodic symptoms for over a decade, worsening in frequency and severity recently.

At age 15, he had a left hemicolectomy with temporary transverse colostomy for presumed Hirschsprung’s disease (HD). Gross examination of the specimen did not show any distal narrowing, and histopathology showed normal ganglion cells, ruling out HD. The patient’s sibling had similar but more severe symptoms and died young due to the condition.

On examination, the patient was afebrile with stable vital signs. The abdomen was soft, nontender, severely distended, tympanic, and without peritonism.

### Diagnostic assessment and intervention

**Figure 1:**
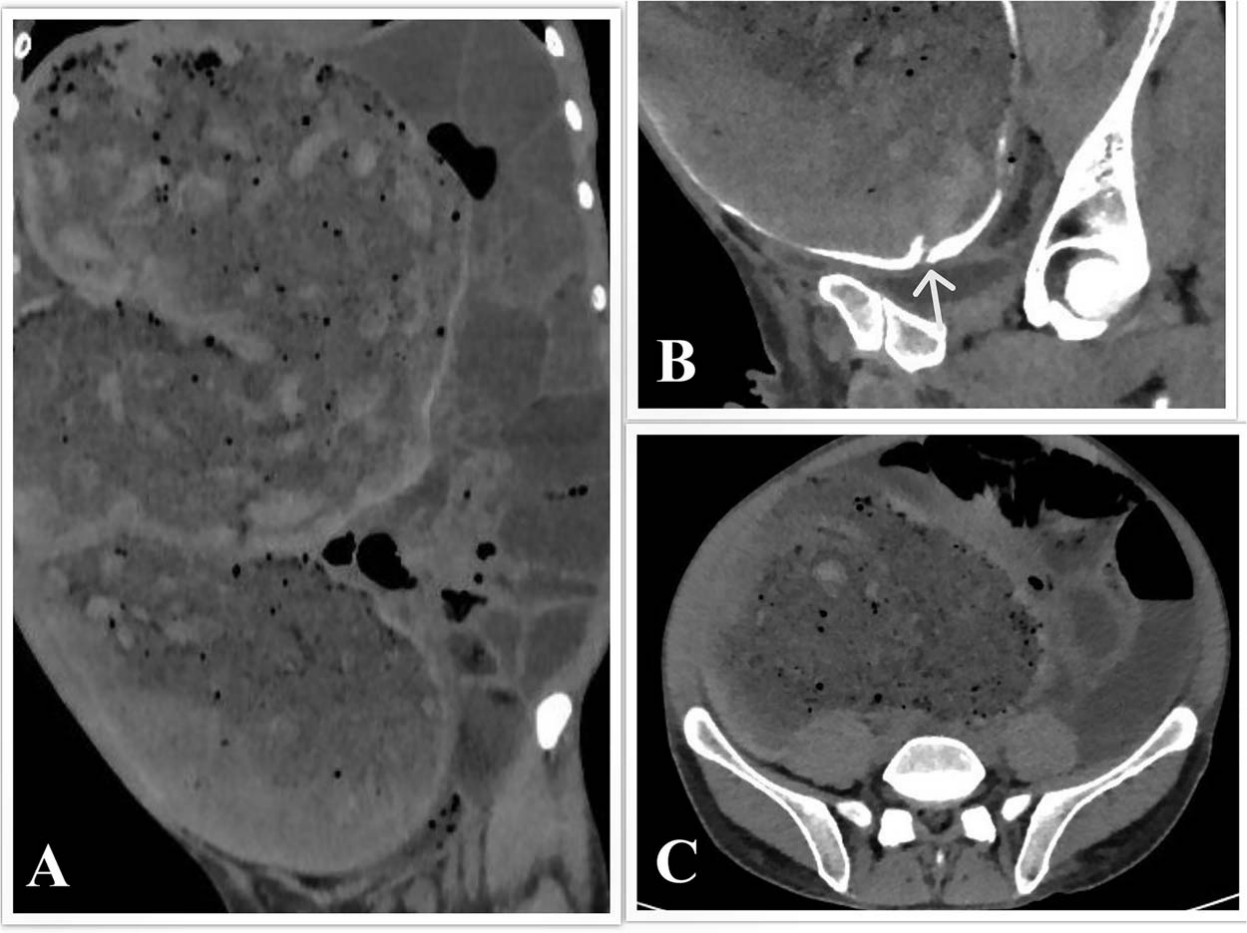
CT scan of the abdomen and pelvis. (A) Coronal view showing the megacolon and small bowel dilatation. (B) Sagittal view showing the sigmoid stricture, with the arrowhead pointing toward it. (C) Axial view showing colon dilatation at the mid-sigmoid level.

Computed tomography (CT) of the abdomen and pelvis with rectal contrast revealed significant fecal loading, colonic dilatation reaching 11.5 cm at the mid-sigmoid level (Fig. 1), a 2 cm stricture 25 cm from the anal verge (Fig. 1B), and small bowel dilatation.

Flexible sigmoidoscopy demonstrated a smooth-walled narrowing in the sigmoid colon without mucosal abnormalities. A guidewire was advanced, and a rectal decompression tube was placed. Suctioning and lavage provided partial and temporary relief. Polyethylene glycol was administered once through the tube, but this was discontinued due to patient discomfort.

Two days later, the patient presented to the emergency department with severe pain and obstipation. Physical examination remained stable. As conservative therapy failed, subtotal colectomy was indicated. Preoperative laboratory studies were unremarkable except for low prealbumin (14.5 mg/dl; normal: 20.0–40.0 mg/dl). Total parenteral nutrition was initiated for optimization.

#### Surgical intervention

**Figure 2:**
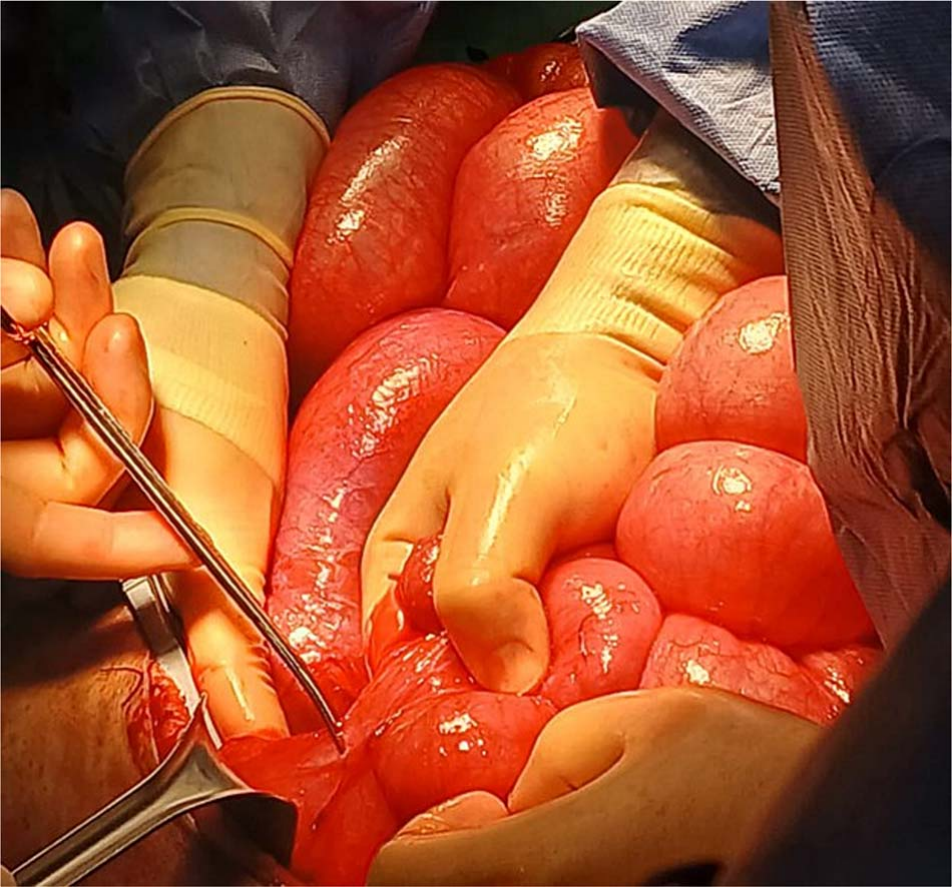
Severe small bowel distension and extensive adhesions.

**Figure 3:**
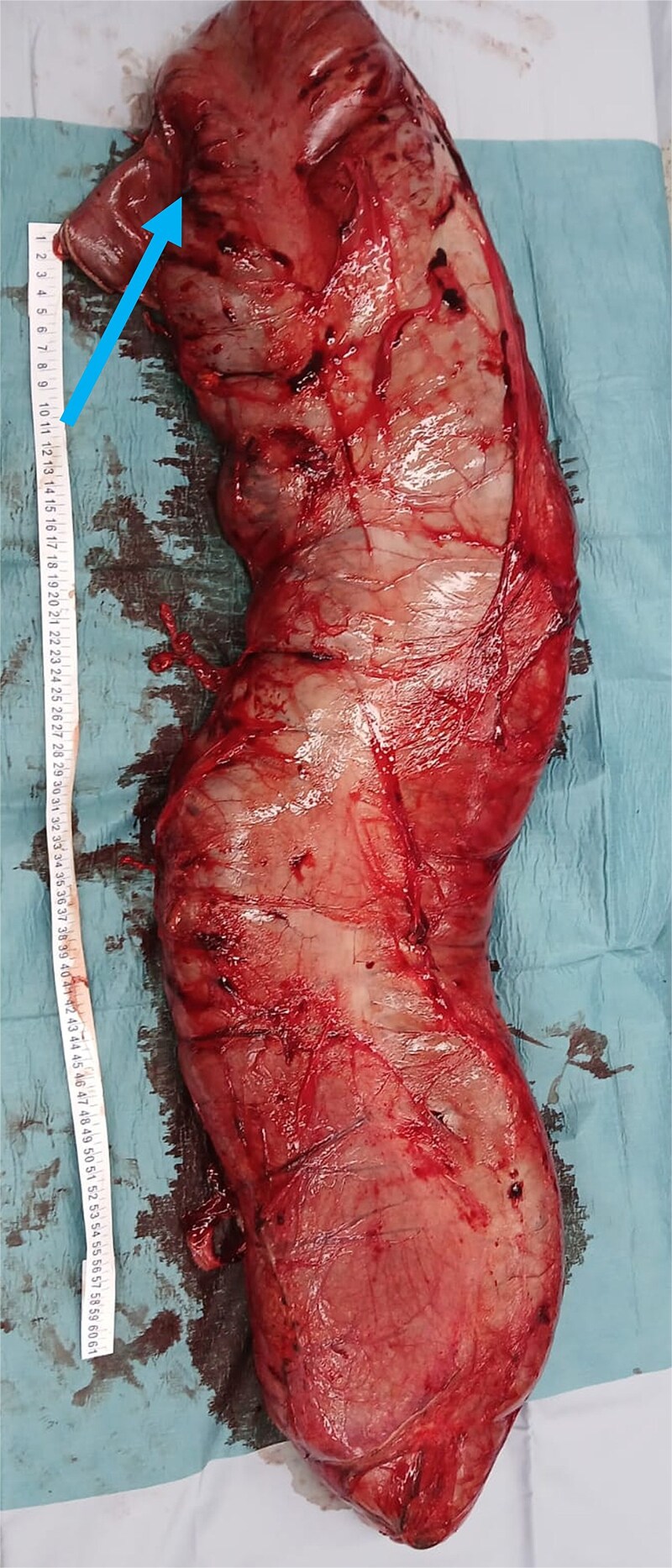
Extracted megacolon. Arrowhead pointing toward the ileocecal valve.

Following intrathecal analgesia and induction of general anesthesia, the patient was placed in a modified Lloyd-Davis position. Midline laparotomy revealed severe small bowel distension and extensive adhesions, which were carefully lysed (Fig. 2). A focal narrowing of the colon was identified at the site of the prior coloproctostomy, with a significantly dilated, stool-filled proximal colon and normal-caliber bowel distal to the anastomosis. The colon was mobilized from the cecum to the rectosigmoid junction using the lateral-to-medial approach, and a subtotal colectomy was completed by dividing the bowel with a linear cutting stapler. The specimen was extracted and sent for histopathology (Fig. 3).

The small bowel was decompressed by milking its contents into a basin, beginning at the duodenojejunal junction. An end-to-side ileorectal anastomosis was fashioned using an end-to-end anastomosis circular cutting stapler. Anastomotic donuts were intact. Leak testing and rigid sigmoidoscopy confirmed anastomotic integrity. A Blake drain was placed, and the abdomen was closed in layers. The patient was extubated 1 hour postoperatively due to mild hypothermia and transferred to the ward.

### Follow-up and outcomes

Postoperative recovery was uneventful. Bowel function resumed on postoperative day (POD) 2, the diet was advanced as tolerated, and the patient was discharged home on POD 4.

The resected specimen measured 95 × 21 × 10 cm (Fig. 3) and weighed 10.5 kg, with a narrowing at the old coloproctostomy site. Histopathology demonstrated the presence of ganglion cells, moderate chronic inflammation, increased eosinophils, and melanosis coli.

At follow-up visits on POD 15 and 30, the patient reported complete symptom resolution and improved quality of life. Bowel movements were irregular but controlled with pharmacotherapy. Ongoing surveillance is symptom-guided.

## Discussion

This case illustrates a rare presentation of chronic idiopathic megacolon in adulthood following previous colectomy, with a possible familial predisposition. Despite earlier surgery, symptoms recurred and progressed, necessitating further resection.

Reoperations for megacolon are uncommon and typically due to residual aganglionosis in Hirschsprung’s disease [12, 13]. Familial adult-onset chronic megacolon has been described in the literature [3]; however, repeat exposure to surgery in such cases is rarely reported.

Preoperative evaluation must rule out HD through a rectal biopsy or by demonstrating an intact anorectal inhibitory reflex [9]. In our case, prior histopathology confirmed ganglion cells, ruling out HD.

Imaging, particularly CT or magnetic resonance imaging, is essential to rule out obstruction [2], measure the colonic diameter, and evaluate for complications such as perforation [9]. A sigmoid diameter > 10 cm is diagnostic [9]; our patient’s reached 11.5 cm. Endoscopy, though typically avoided for perforation risk [9], can aid in diagnosis, biopsy [2], and decompression [14], as in this case.

Subtotal colectomy with ileorectal anastomosis remains the treatment of choice for refractory idiopathic megacolon, offering favorable outcomes [15]. In malnourished or high-risk patients, diversion may be considered [15]. After nutritional optimization, our patient underwent primary anastomosis with excellent results.

Histopathology confirmed idiopathic megacolon with preserved ganglion cells. Melanosis coli likely reflected chronic laxative use, while eosinophilic infiltration suggested reactive inflammation [16].

The positive family history supports a genetic myopathy/neuropathy [7, 8]. A pathogenic SEMA3F mutation has been found in a familial cluster [3]. Although genetic testing was unavailable, it may clarify diagnosis, inform counseling, and guide future research.

Postoperatively, adherence to Enhanced Recovery After Surgery protocols and gradual diet advancement are essential. Absence of the ileocecal valve may predispose to diarrhea, necessitating careful management to prevent dehydration.

## Conclusion

This report underscores the rarity of adult-onset idiopathic megacolon and its potential familial component. Idiopathic megacolon should be considered in adults with chronic colonic dilatation once secondary causes have been excluded. Comprehensive family history and, where available, genetic testing are important to identify hereditary predisposition.

Subtotal colectomy with ileorectal anastomosis is a safe and effective option for patients with refractory symptoms, providing significant symptomatic relief and functional improvement.

The limitations of this report include its single-patient nature, which restricts generalizability, and the absence of genetic analysis, which precludes definitive etiologic identification.
